# Gestational age at birth in pregnancies with antenatal corticosteroid administration in relation to risk factors: a retrospective cohort study

**DOI:** 10.3389/fmed.2023.1285306

**Published:** 2024-01-09

**Authors:** Joachim Graf, Harald Abele, Jan Pauluschke-Fröhlich

**Affiliations:** ^1^Midwifery Science, Institute for Health Sciences, University Hospital Tübingen, Tübingen, Germany; ^2^Department of Women’s Health, University Hospital Tübingen, Tübingen, Germany

**Keywords:** premature birth, gestational age at birth, AIS, extreme immaturity, PPROM

## Abstract

**Background:**

The aim was to investigate gestational age at birth of women after induction of fetal lung maturation (antenatal corticosteroids = ACS) because of imminent preterm birth (PTB), and to quantify incidence of late PTB (gestational age < 260 days) and extreme immaturity (gestational age < 196 days) in relation to several diagnoses (PPROM, placental bleeding, premature labor, preeclampsia, oligohydramnios, amniotic infection syndrome (AIS), cervical insufficiency) and risk factors (age > 35, history of preterm delivery, multifetal gestation, gestational diabetes, hypertension, nicotine abuse).

**Methods:**

The study was designed as a retrospective cohort trial, in which the data of all births taking place in 2016 in the German federal state Rhineland-Palatinate were evaluated. Frequency analyses, subgroup analysis (Chi-square tests and Friedman’s tests), as well as multinomial logistic regressions and linear regressions were used to determine odds ratios (OR).

**Results:**

In total, *N* = 1,544 patients were included who had been hospitalized due to an imminent PTB and had received ACS, of whom 52% had a late PTB and 8% a PTB with extreme immaturity. Regarding the gestational age at birth, there were only minor differences between the individual risk factors and diagnoses, only AIS patients showed a significantly lower gestational age (mean: 207 days). A significantly increased risk of PTB with extreme immaturity was found in patients with AIS (OR = 5.57) or placental bleeding (OR = 2.10).

**Conclusion:**

There is a need for further research in order to be able to apply therapeutic measures more accurately in relation to risk factors and diagnoses.

## 1 Introduction

With a prevalence of about 8%, prematurity is one of the most common pregnancy-related problems in Germany (1). Preterm birth (PTB) is defined as a birth event before the 260th day of pregnancy (<37th week of pregnancy), while PTB with extreme immaturity is defined as delivery before the 196th day (<28th week of pregnancy) (2, 3). Of the numerous factors contributing to the increased risk of PTB are maternal age > 35 years (4), history of a previous preterm delivery, stillbirth or miscarriage in personal anamnesis (5, 6), gestational diabetes or hypertension (6), nicotine abuse (7), as well multiple pregnancies (8, 9). Women with the diagnoses preterm premature rupture of membranes (PPROM), placenta bleeding, premature labor, preeclampsia, oligohydramnios, amniotic infection syndrome (AIS) or cervical insufficiency also show a higher risk of PTB (6, 10–12). If symptoms of any of these diagnoses occur before the 34th week of pregnancy, induction of fetal lung maturation (Antenatal corticosteroids, ACS) should be performed to prevent infant respiratory distress syndrome (IRDS) (13). IRDS is reported in 60% of all PTBs before the 30th week of pregnancy and it is the most common cause of death in these babies (14), while ACS administration reduces the risk of IRDS as well as morbidity and mortality in preterm neonates (15, 16). From the perspective of women’s health research, it is of relevance how often PTB occurs in women classified as at risk of PTB, and under which conditions PTB ensues either before the 196th day or (more likely) before the 260th day of pregnancy. Since the clinical effect of ACS is only proven within a very narrow time window (>24 h, <7 days), it is important that the timing between administration and actual birth is as precise as possible (17, 18). To increase the efficiency of clinical interventions, more epidemiological studies are needed to show how often a premature birth event occurs when certain risk factors are present. Against this background, the aim of this study was to investigate gestational age at birth of women after ACS because of imminent PTB, and to quantify the incidence of late PTB and extreme immaturity in relation to several previously diagnosed pathologies (PPROM, placental bleeding, premature labor, preeclampsia, oligohydramnios, AIS, cervical insufficiency) and risk factors (age > 35, history of preterm delivery, stillbirth or abortion in personal anamnesis, multifetal gestation, gestational diabetes, hypertension, nicotine abuse).

## 2 Materials and methods

### 2.1 Study design and data management

In total, *N* = 1,544 patients were included who had been hospitalized due to an imminent PTB and had received ACS in 2016. All patients whose symptoms and risk profiles did not lead to induction of fetal lung maturation were excluded. The study was designed as a retrospective cohort trial. The birth data of the Geschäftsstelle Qualitätssicherung Rheinland Pfalz (SQMed RLP = Quality Assurance Office Rhineland-Palatinate) was requested, which manages the data of all births taking place in the German federal state of Rhineland-Palatinate (about 38,000 births per year). The study population was classified into eight subgroups of diagnosis (PPROM, placental bleeding, premature labor, preeclampsia, oligohydramnios, AIS, cervical insufficiency and unknown referral diagnosis) and the risk profile was determined. The risks defined were maternal age > 35 years, history of preterm delivery, stillbirth or miscarriage in personal anamnesis (calculated from the difference between gravidities and parities number), multiple pregnancies, gestational diabetes, hypertension and nicotine abuse.

### 2.2 Statistics

First, frequency analyses were performed. Subgroup analysis was then conducted to compare patients with and without premature birth using both Chi-square tests (Fisher’s exact test) and Friedman’s tests. Chi-square tests were also performed to compare patients with premature birth and extreme immaturity, as well as multinomial logistic regressions (for the non-dichotomous variables) and linear regressions (for the binary coded variables) were used to determine the odds ratio (OR) in relation to diagnosis/ACS indicators and PTB risk factors. In all analyses, value of *p*s <0.05 (two-tailed) were considered indicative of statistically significant differences (*α* = 0.05). All statistical analyses were conducted using IBM SPSS Statistics (version 24).

## 3 Results

### 3.1 Gestational age in relation to PTB causes and risk factors

**Figure 1:**
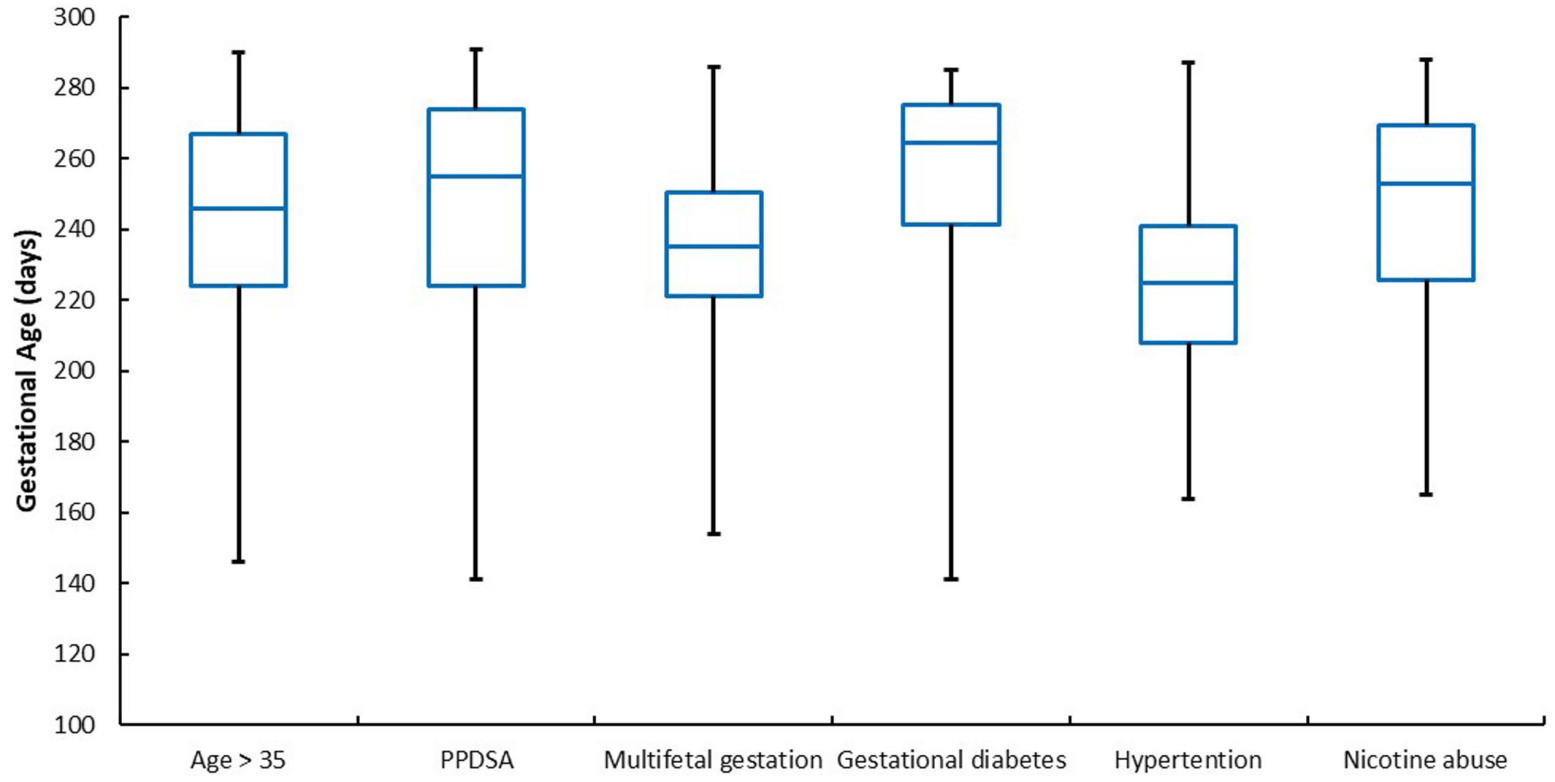
Gestational age at birth in relation to PTB risk factors, *n* = 1.544. *p* = < 0.001 (Friedman test); PPDSA, previous preterm delivery, stillbirth or abortion.

**Figure 2:**
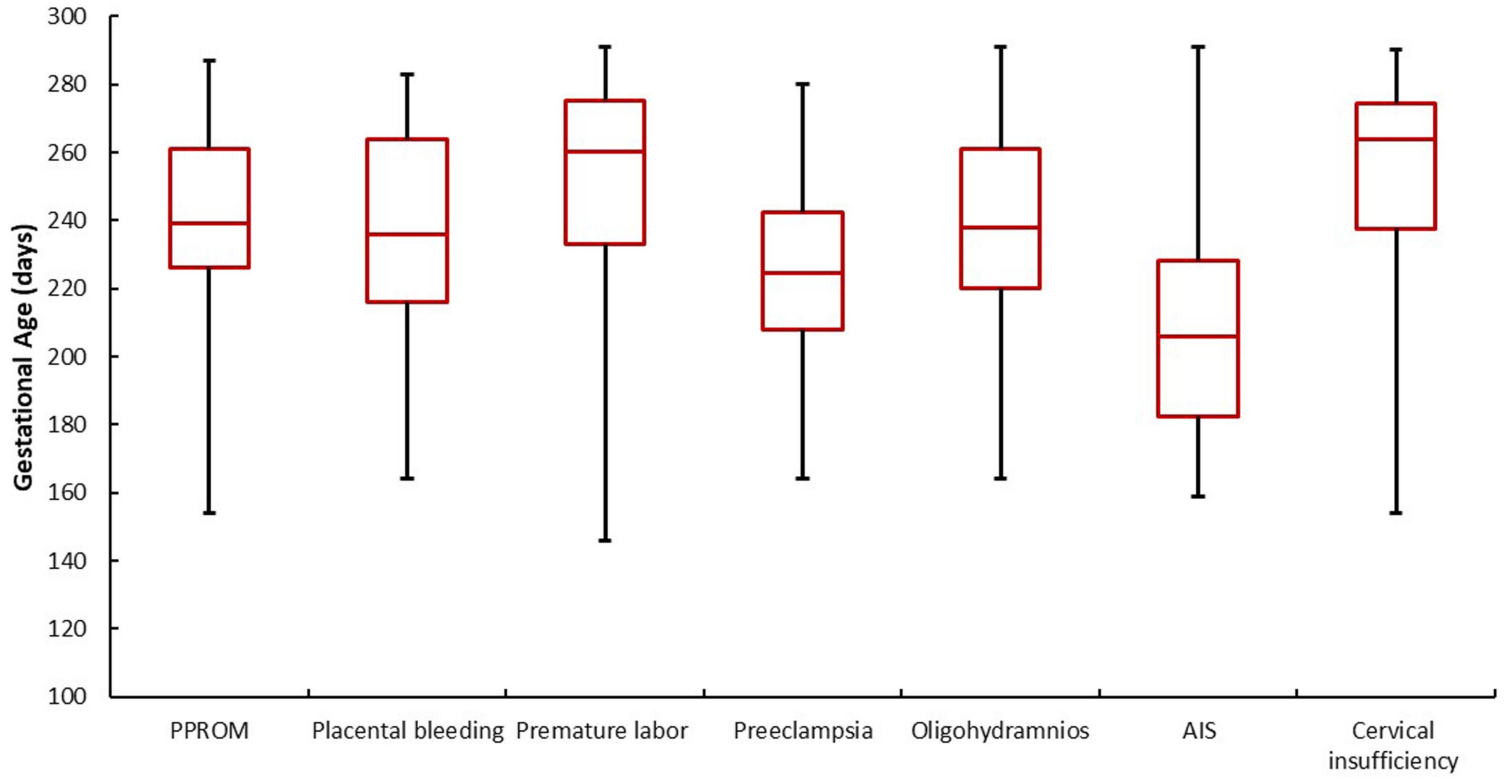
Gestational age at birth in relation to ACS causes, *n* = 1.544. *p* = < 0.001 (Friedman test); AIS, amniotic infection syndrome; PPROM, preterm premature rupture of membranes.

With regard to the PTB risk factors, there were differences in gestational age, as this was on average (median) in the range between 225 and 264 days of pregnancy (Figure 1). However, a different picture emerged for the diagnoses, as shown in Figure 2, since the range was significantly larger depending on the diagnosis (medians): gestational age at birth varied between 206 days and 264 days. In relation to the diagnosis groups, the lowest gestational age was found in women diagnosed with AIS (206 days), while the average gestational age was 225 days for preeclampsia, 236 days for placental bleeding, 238 days for oligohydramnios, 239 days for PPROM, 260 days for preterm labor and 264 days for cervical insufficiency.

### 3.2 PTB risk in relation to risk factors and diagnoses

The comparison of women who received ACS and actually had a preterm birth (*n* = 925, Group 1) and those who received ACS without preterm birth (*n* = 619, Group 2) showed significant differences with a lot diagnoses and risk factors. Group 1 patients were significantly older (women >35 years) and showed a higher proportion of multifetal gestation and hypertension as risk factors as well as a higher proportion of PPROM, placental bleeding, preeclampsia, oligohydramnios and AIS. In contrast, the proportion of women with a history of preterm delivery, stillbirth or abortion in personal anamnesis in their personal anamnesis was significantly decreased in this group as well as proportion of premature labor and cervical insufficiency (Table 1).

**Table 1:** Sociodemographics, PTB risk factors and ACS diagnoses in the overall population.

Variable	ACS patients with premature birth (*n* = 925, 59.9%)	ACS patients without premature birth (*n* = 619, 40.1%)	*p*-value
Age (years)			<0.0001*
Mean (SD)	30.95 (5.35)	29.38 (5.86)	
Median (Min; Max)	31 (16;48)	29 (14; 47)	
Gestational age (days)			<0.0001*
Mean (SD)	225.99 (23.59)	272.95 (7.78)	
Median (Min; Max)	231 (141; 259)	273 (260; 293)	
PTB risk factors			
Age > 35	181 (19.57%)	104 (16.80%)	0.170
History of preterm delivery, stillbirth or abortion in personal anamnesis	265 (28.65%)	216 (34.89%)	0.009*
Multifetal gestation	199 (21.51%)	28 (4.52%)	<0.0001*
Gestational diabetes	9 (0.97%)	13 (2.10%)	0.067
Hypertension	64 (6.92%)	13 (2.10%)	<0.0001*
Nicotine abuse	78 (8.43%)	62 (10.02%)	0.288
ACS diagnoses			
PPROM	394 (42.59%)	141 (22.78%)	<0.0001*
Placental bleeding	93 (10.05%)	42 (6.79%)	0.025*
Premature labor	205 (22.16%)	222 (35.86%)	<0.0001*
Preeclampsia	119 (12.86%)	17 (2.75%)	<0.0001*
Oligohydramnios	103 (11.14%)	42 (6.79%)	0.004*
AIS	63 (6.81%)	3 (0.48%)	<0.0001*
Cervical insufficiency	112 (12.11%)	151 (24.39%)	<0.0001*
None of the current diagnoses	176 (19.03%)	172 (27.79%)	<0.0001*
>1 diagnosis	283 (30.59%)	143 (23.10%)	0.001*

*Statistically significant at *p* < 0.05 in Chi-square tests (Fisher’s exact test) and Friedman’s tests. ACS, antenatal corticosteroids; AIS, amniotic infection syndrome; PPROM, preterm premature rupture of membranes; PTB, preterm birth.

### 3.3 OR for late PTB and extreme immaturity in relation to PTB risk factors and ACS diagnoses

In the study population, which consisted of all patients at risk for PTB and received ACS, there were *n* = 809 patients (52%) with late PTB (gestational age < 260 days and ≥ 196 days) and *n* = 116 (8%) newborns with extreme immaturity (gestational age < 196 days). Forty percent were born at term (gestational age > 260 days). For clinical management, it is important to examine to what extent individual risk factors model the risk of a PTB with extreme immaturity or a late PTB. A significantly increased risk of PTB with extreme immaturity was observed in patients with AIS (OR = 5.57, *p* = <0.0001) and placental abruption (OR = 2.10, *p* = 0.012). Maternal age modulated this risk with OR = 1.18 (*p* = <0.0001). This means that in patients after induction of fetal lung maturation, the OR for extreme preterm birth increases by an average of 18% with each additional year of life. There were no significant differences for all other risk factors and diagnoses in OR, so all were equally likely to cause either late PTB or extreme immaturity PTB (Table 2).

**Table 2:** OR for late PTB and PTB with extreme immaturity in relation to PTB risk factors and ACS diagnoses.

Variable	Late PTB (Gestational age <260 days; *n* = 809)	PTB with extreme immaturity (Gestational age < 196 days; *n* = 116)	*p*-value	OR	95%-CI
Age (years)					
Mean (SD)	31.01 (5.35)	30.55 (5.34)	<0.0001**	1.18	[1.05; 1.31]
Median (Min; Max)	31 (16; 48)	30 (18; 44)			
PTB risk factors					
Age > 35	159 (19.65%)	22 (18.97%)	>0.99	0.96	[0.58; 1.57]
History of preterm delivery, stillbirth or abortion in personal anamnesis	223 (27.56%)	42 (36.21%)	0.062	1.49	[0.99; 2.25]
Multifetal gestation	179 (22.13%)	20 (17.24%)	0.28	0.73	[0.44; 1.22]
Gestational diabetes	8 (0.99%)	1 (0.86%)	>0.99	0.87	[0.11; 7.03]
Hypertension	57 (7.05%)	7 (6.42%)	0.85	0.85	[0.38; 1.91]
Nicotine abuse	64 (7.91%)	14 (7.24%)	0.15	1.60	[0.86; 2.95]
ACS diagnoses					
PPROM	351 (43.39%)	43 (37.07%)	0.23	0.77	[0.51; 1.15]
Placental bleeding	73 (9.02%)	20 (17.24%)	0.012*	2.10	[1.23; 3.60]
Premature labor	178 (22.00%)	27 (23.28%)	0.81	1.08	[0.68; 1.71]
Preeclampsia	107 (13.23%)	12 (10.34%)	0.46	0.76	[0.40; 1.42]
Oligohydramnios	92 (11.37%)	11 (9.48%)	0.64	0.82	[0.42; 1.58]
AIS	25 (3.09%)	38 (32.76%)	<0.0001*	5.57	[3.22; 9.66]
Cervical insufficiency	99 (12.24%)	13 (11.21%)	0.88	0.91	[0.49; 1.67]
>1 diagnosis	243 (40.04%)	40 (34.48%)	0.085	1.23	[0.97; 1.55]
Gestational age (days)					
Mean (SD)	232.73 (16.02)	179.02 (11.09)	<0.0001*		
Median (Min; Max)	234 (169; 259)	181.5 (141; 195)			

*Statistically significant at *p* < 0.05 in linear regression.

**Statistically significant at *p* < 0.05 in multinomial logistic regression.

ACS, antenatal corticosteroids; AIS, amniotic infection syndrome; CI, confidence interval; OR, Odds Ratio; PPROM, preterm premature rupture of membranes; PTB, preterm birth.

## 4 Discussion

### 4.1 Principal results and limitations

We found that in patients at risk of PTB, a significantly lower gestational age can be expected in cases of existing AIS and placental bleeding, while all other diagnoses and risk factors had only small effects on the average gestational age. In the total collective, 52% had late PTB and 8% PTB with extreme immaturity, which means that 40% of all patients did not have a PTB after ACS management. Patients with a history of preterm delivery, stillbirth or miscarriage in their personal anamnesis also had an increased risk of having a PTB with extreme immaturity, although that effect was slightly non-significant (*p* = 0.062). A relevant limitation of this study is that no data were available on pre-existing morbidity and additional therapeutic measures such as uterine contraction inhibitors (19) or cerclages in patients with cervical insufficiency (20). We assume that the comparatively high average gestational duration time in patients with cervical insufficiency is due to the fact that treatment was carried out in accordance with the guideline recommendation (e.g., via pessary). A further limitation is that not all clinically relevant risk factors for PTB were included in the data set, as, e.g., polyhydramnios and *in vitro* fertilization are also associated with an increased risk of preterm birth (21, 22). Furthermore, it was not possible to find out how often and how many doses of ACS were administered. The cohort analysis did not cover all women with an increased risk of preterm birth, since data were only available for patients who were treated with ACS. The examined collective included only pregnancies after induction of fetal lung maturity. Since most preterm births represent late preterm births (>34 weeks of pregnancy), the study collective represented only 4.12% of all births in Rhineland-Palatinate [37,518 births in 2016 (23)]. Since only 925 women in the study collective actually suffered a preterm birth, this rate was only 2.47% of all births and thus only about a quarter of the expected preterm births. Critically, all preterm births before 34 weeks of pregnancy that did not receive ACS could not be considered, which makes the exact identification of risk collectives more difficult. The strengths of the present study include the availability of a full-year cohort of ACS-treated patients, which enhances knowledge about specific risk collectives. In cases of extreme preterm birth, other measures such as neuroprotection should be considered in addition to ACS induction (24). There are indications that this is especially true for patients with AIS and placental bleeding. The literature also corroborates that especially in patients with AIS, a very rapid birth event and thus a low gestational age can be expected (25, 26). A further limitation resulted from the fact that premature births, stillbirths and (voluntary) abortions in the personal medical history had to be considered as a common risk factor in this study. This was due to the fact that data from the statutory quality monitoring system was evaluated, in which no differentiation was made between individual parameters. The resulting confounding could possibly have led to a higher proportion of patients with the corresponding risk factor being in the group in which no PTB event occurred. It is known from the literature that both stillbirths (27) and condition after premature birth (28) increase the risk of PTB, while the influence of induced abortion on the risk of PTB is controversial (29, 30). In the data provided by the quality monitoring, no differentiation was made between spontaneous PTB and medically induced PTB, which is why the authors are unable to make any statement on this, which has a corresponding limiting effect. However, there are no comparative studies in which different risk collectives are compared in terms of gestational age in a comparably large collective. It is known that the risk of PTB with extreme immaturity increases in the case of several existing risk factors (31). In these studies, the focus was on the patients who did give birth and not on the patients who did not give birth despite existing risks. Since the majority of women did not give birth prematurely despite individual preterm birth risks, an analysis of this group in particular would be very interesting in order to prevent unnecessary lung maturation.

### 4.2 Implications for practice and/or policy

For defined risk factors, women at increased risk of preterm birth are given ACS until the 34th week of pregnancy to accelerate fetal lung maturity. As the clinical effect of ACS has only been demonstrated within a very narrow time window (>24 h, <7 days), it is important that the timing between administration and actual delivery is as precise as possible (17, 18). The present study results can support practice in deciding whether or not to administer ACS, as corticosteroids have side effects on the child’s long-term development and therefore the indication for lung maturation needs to be made carefully (32).

### 4.3 Conclusion

Preterm birth is one of the common pregnancy-related risks. In particular, a PTB at gestational age < 196 days is linked to high fetal morbidity and mortality. Although the causes and risk factors can be described as well-researched, too little attention has been paid to how gestational age differs depending on diagnoses and risk factors in the context of ACS management. There is a need for further research to be able to carry out therapeutic measures more accurately. Significantly, a PTB occurred in 60% of patients in our study group who received ACS. Among patients considered to be at particular risk for PTB, there are no differences in terms of average gestational age at birth relative to most risk factors and diagnoses. In patients suffering from AIS and placental bleeding, there is a significantly higher risk of PTB with extreme immaturity, which is why these patients could benefit not only from the administration of ACS but also, for example, from neuroprotection.

## Data availability statement

The raw data supporting the conclusions of this article will be made available by the authors, without undue reservation.

## Ethics statement

Ethical approval was not required for the study involving humans in accordance with the local legislation and institutional requirements. Written informed consent to participate in this study was not required from the participants or the participants’ legal guardians/next of kin in accordance with the national legislation and the institutional requirements.

## Author contributions

JG: Methodology, Validation, Visualization, Writing – original draft. HA: Validation, Writing – review & editing. JP-F: Conceptualization, Project administration, Supervision, Writing – original draft, Writing – review & editing.
